# Party Balloon Inflation Maneuver During Saline Contrast Transthoracic Echocardiography to Detect Patent Foramen Ovale

**DOI:** 10.1016/j.jaccas.2021.10.012

**Published:** 2022-01-19

**Authors:** Akihisa Kataoka, Kento Kito, Takeyuki Sajima, Yusuke Watanabe, Ken Kozuma

**Affiliations:** a Department of Medicine, Division of Cardiology, Teikyo University, Tokyo, Japan; b Department of Anesthesia, Teikyo University, Tokyo, Japan

**Keywords:** bubble echocardiography, congenital heart defect, echocardiography, stroke, PFO, patent foramen ovale, TEE, transesophageal echocardiography, TTE, transthoracic echocardiography

## Abstract

Saline contrast echocardiography requires an adequate provocation method for the detection of patent foramen ovale. The party balloon inflation maneuver during saline contrast transthoracic echocardiography is easy to explain to patients and objectively assesses the performance of provocative maneuvers by a clinician by watching balloon inflation. (**Level of Difficulty: Intermediate.**)

## Central Illustration

**Figure:**
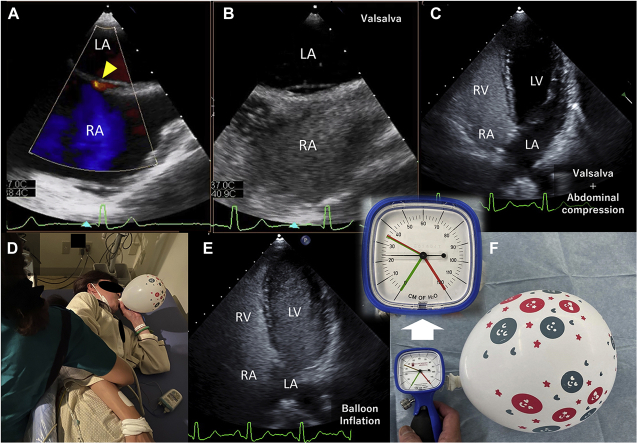

**Figure 1:**
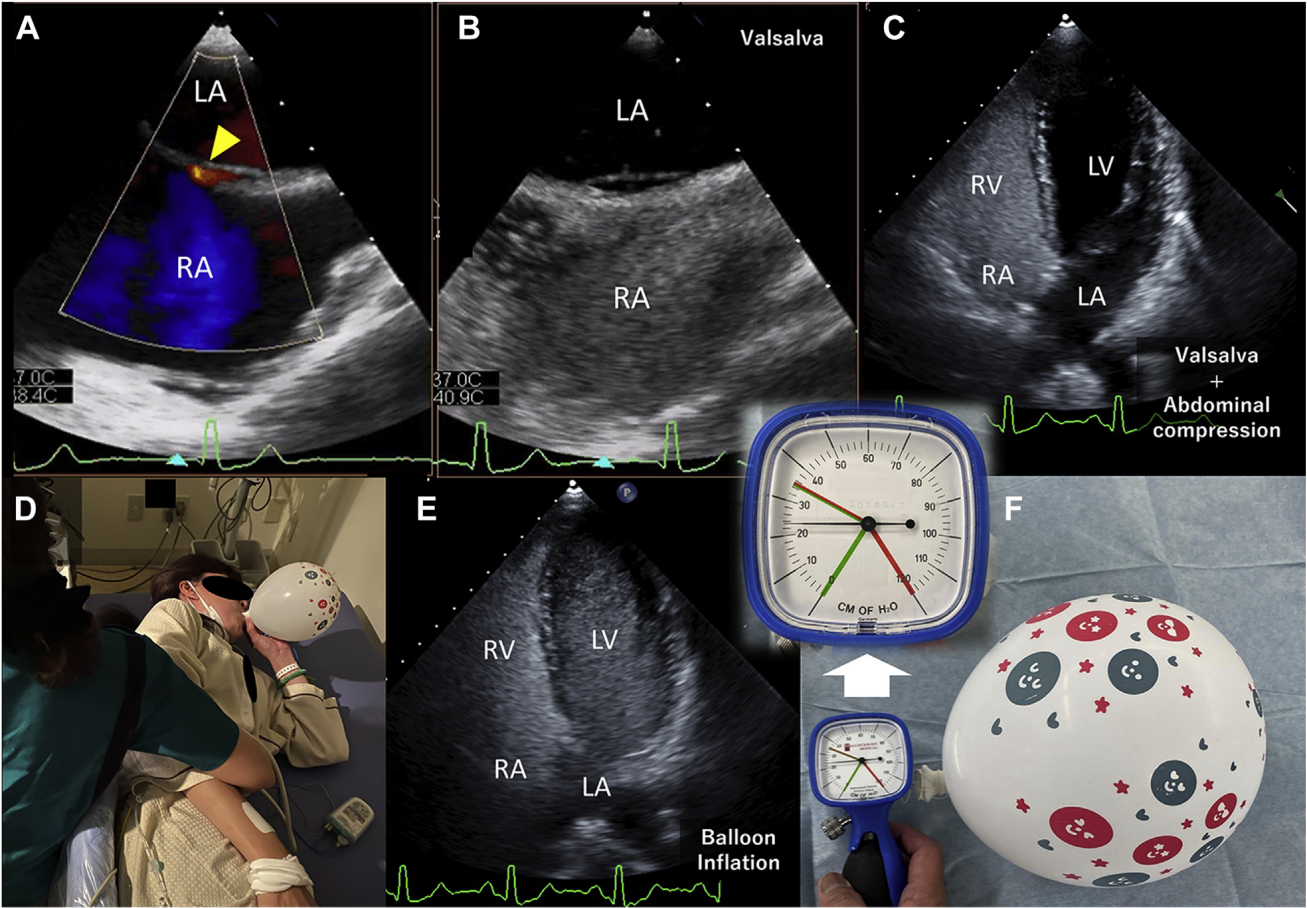
Transesophageal Echocardiography and Transthoracic Echocardiography Images, Party Balloon Inflation Maneuver, and Pressure Measurement Color Doppler transesophageal echocardiography images with an omniplane angle of 120° **(A)** and saline contrast transesophageal echocardiography with an omniplane angle of 120° **(B)** during spontaneous Valsalva maneuver. Saline contrast transthoracic echocardiography images during spontaneous and abdominal compression Valsalva maneuver **(C)** and balloon inflation maneuver **(E)**. Patient performs balloon inflation maneuver **(D)**. Pressure measurement in the dry laboratory **(F)**. The white arrow shows an enlarged view of the face of the cuff manometer during balloon inflation. LA = left atrium; LV = left ventricle; RA = right atrium; RV = right left ventricle.

A 45-year-old woman with a history of cryptogenic stroke was referred to us for consultation. Transesophageal echocardiography (TEE) showed that the patent foramen ovale (PFO) had a left-to-right shunt (Figure 1A, yellow triangle, Video 1). However, there was no evidence of an obvious right-to-left shunt due to inadequate Valsalva maneuvers because the patient was sedated (Figure 1B, Video 2). Therefore, saline contrast transthoracic echocardiography (TTE) was performed on another day. However, repeated saline contrast TTEs with spontaneous and abdominal compression during Valsalva maneuver did not show microbubbles in the left chambers (Figure 1C, Video 3). Instead of conventional provocative maneuvers, a party balloon (Daiso Industries Co, Ltd) was inflated during saline contrast injection (Figure 1D). TTE results revealed a continued microbubble opacification in the left chambers on one cardiac cycle without balloon deflation (Figure 1E, Video 4), indicating the presence of a right-to-left shunt through the PFO. Therefore, the patient underwent transcatheter PFO closure without any residual shunts.

The experiment in the dry laboratory revealed that filling the party balloon with air added 20 to 25 mm Hg positive end-expiratory pressure to the patient’s airway (Figure 1F), which increased the intrathoracic pressure and impeded the blood return. With inspiration to further inflate the party balloon, the intrathoracic pressure decreased to negative and there was an increased return of blood to the right atrium, and a right-to-left atrial pressure gradient was generated (1,2).

PFOs are dynamic entities. The proof of a right-to-left shunt is mandatory before transcatheter PFO closure. Saline contrast TEE remains the standard reference to detect right-to-left shunt, but TEE is semi-invasive and is not ideal for screening. In contrast, saline contrast TTE is noninvasive and can be used for screening (3). However, the optimal methodologies of saline contrast TTE for the detection of PFO, which requires an adequate provocation method, have not been established. In this case, the Valsalva maneuver was not correctly performed, but the use of the party balloon worked because it was simple to explain to the patient and it provided visual feedback that the maneuver had been performed correctly.

In conclusion, this maneuver is easy to explain to patients and objectively assesses the performance of provocative maneuvers by a clinician by observing balloon inflation. Hence, this may be beneficial over standard provocative methods and may be a potentially helpful addition to the workflow for busy echo labs using a simple tool.

## Funding Support and Author Disclosures

The authors have reported that they have no relationships relevant to the contents of this paper to disclose.

## Appendix

Supplemental Video 1 Simultaneous biplane color Doppler image of TEE showing the PFO with left-to-right shunt.

Supplemental Video 2 Saline contrast simultaneous biplane moving image of the TEE during spontaneous Valsalva maneuver. There were no considerable microbubbles. However, a few bubbles in the left atrium are seen (shunt grade 1). This suggests that there is no presence of the right-to-left shunt for invasive therapy.

Supplemental Video 3 Saline contrast TTE moving image with spontaneous and abdominal compression Valsalva maneuver. The TTE does not demonstrate microbubbles in the left chambers.

Supplemental Video 4 Saline contrast TTE moving image with balloon inflation maneuver. Microbubble opacification in the left chambers during one cardiac cycle without balloon deflation is shown. This indicates the presence of the right-to-left shunting through the PFO (shunt grade 4).
